# Novel Epitope Mapping of African Swine Fever Virus pI215L Protein Using Monoclonal Antibodies

**DOI:** 10.3390/v15102081

**Published:** 2023-10-12

**Authors:** Yanni Gao, Xiaolin Jiang, Xing Yang, Keshan Zhang, Ping Jiang, Juan Bai

**Affiliations:** 1 Key Laboratory of Animal Diseases Diagnostic and Immunology, Ministry of Agriculture, MOE Joint International Research Laboratory of Animal Health and Food Safety, College of Veterinary Medicine, Nanjing Agricultural University, Nanjing 210095, China; 2 State Key Laboratory of Veterinary Etiological Biology, College of Veterinary Medicine, Lanzhou Veterinary Research Institute, Chinese Academy of Agricultural Sciences, Lanzhou University, Lanzhou 730046, China; 3 Jiangsu Co-Innovation Center for the Prevention and Control of Important Animal Infectious Diseases and Zoonoses, Yangzhou University, Yangzhou 225009, China

**Keywords:** African swine fever virus, pI215L protein, monoclonal antibodies, B cell epitope

## Abstract

The African swine fever virus (ASFV) is one of the most important pathogens that causes huge damage to worldwide swine production. The pI215L protein is found within the virion and expressed at a high level in infected porcine alveolar macrophages (PAMs), indicating a possible role of pI215L protein in ASFV detection and surveillance. In the present study, female BALB/c mice (5–6-week-old) were immunized with rpI215L protein, and six hybridomas, 1C1, 2F6, 2F10, 3C8, 5E1 and 5B3, steadily secreted anti-pI215L monoclonal antibodies (mAbs). Among them, 1C4, 5E1, and 5B3 had the IgG1 isotype with a Lambda light chain, 2F10 and 3C8 had the IgG1 isotype with a Kappa light chain, and 2F6 had the IgG2a isotype with a Kappa light chain. Western blot showed a good reactivity of the six mAbs against ASFV. Eight truncated polypeptides were produced for epitope mapping. Two novel B cell epitopes, 67LTFTSEMWHPNIYS80 and 167IEYFKNAASN176, were identified by the mAbs. Further analysis revealed that 2F6 mAb could be widely used in ASFV surveillance and 5B3 mAb might serve as a tool in the distinguishment of different ASFV genotypes. This study provides tools of monoclonal antibodies for further study of I215L function and contributes to the development of serological diagnosis and vaccine research.

## 1. Introduction

African swine fever (ASF) is an acute, febrile, and highly contagious disease of domestic pigs caused by the African swine fever virus (ASFV), with an acute infection mortality as high as 100% [1]. ASF is endemic in Sub-Saharan Africa [2], but since its introduction to the Caucasus region in 2007, a highly virulent strain of ASFV has continued to circulate and spread into Eastern Europe and Russia, and most recently into Western Europe and Asia [3]. Due to its fast spread, ASFV is causing a seriously increasing economic impact on the pig industry. Currently, ASF control is mainly dependent on early diagnosis and elimination of infected animals as there are still few worldwide recognized safe and effective vaccines or drugs available to prevent ASFV outbreaks [4]. Therefore, a highly accurate and sensitive diagnostic approach with appropriate targets against ASFV is of great significance for ASF prevention and control.

ASFV is a large, enveloped virus with icosahedral morphology and belongs to the Asfarviridae family, isolates of which have linear dsDNA genomes of 170–194 kbp, encoding more than 150 polypeptides [5]. Monoclonal antibodies against various ASFV proteins, including the major capsid protein p72, the viral internal envelope protein p30 and p17, the outer envelope protein CD2v, the type II transmembrane protein p54, the nonstructural protein pK205R, and the uncharacterized protein pCP312R and pC129R, have been generated for ASFV infection research and development of ASFV diagnosis [6,7,8,9,10,11,12,13]. pI215L, a nonstructural protein of ASFV, is found within the virion and expressed at a high level in infected porcine alveolar macrophages (PAMs) from 4 h post-infection (hpi) [14,15], indicating a possible role of pI215L protein in the early steps of infection. pI215L protein is the only known ubiquitin-conjugating enzyme encoded by a virus, and its activity is essential for ASFV infection as the knockdown of this gene by siRNA impaired viral infection [16]. pI215L protein not only acts as an immunomodulator by impairing NF-κB activation and inhibiting IFN-I signaling [14,17,18] but also regulates host and viral protein synthesis by interacting with host translation machinery [16,19]. However, little is known about the antigenic characteristics of the pI215L protein.

In this study, six monoclonal antibodies (mAbs) against ASFV pI215L protein were generated, and two novel B cell epitopes were identified. Antigenic characteristics and sequence conservation analysis of the epitopes suggested that the pI215L protein could serve as a useful target during ASF surveillance. This study will contribute to the development of ASFV immunodiagnostic research and the functional study of ASFV pI215L protein.

## 2. Materials and Methods

### 2.1. Cells, Virus, Serum and Animals

Mouse myeloma cells SP2/0 were maintained in Roswell Park Memorial Institute (RPMI)-1640 medium that was supplemented with 10% heat-inactivated fetal bovine serum (FBS) with 1% streptomycin and penicillin, and 1% L-glutamine. PAMs were maintained in the supplemented RPMI-1640 medium including 10% porcine serum and 1% streptomycin and penicillin. ASFV genotype II strain CN/GS/2018, which was isolated with PAMs at the Lanzhou Veterinary Research Institute, was used in the present study. Anti-ASFV antibody positive (obtained from the ASFV-infected pigs) and negative serum were kindly provided by Lanzhou Veterinary Research Institute, Chinese Academy of Agricultural Sciences. BALB/c, 5- to 6-week-old, female, specific pathogen-free mice were purchased from Yangzhou University Experimental Animal Center.

### 2.2. I215L Recombinant Plasmid Construction

*I215L* gene sequence was retrieved from the GeneBank database (GenBank: MK333180.1) and amplified with pI215L-PF and -PR primers (Table 1). The amplicon was cloned into pET28a using T4 DNA ligase (New England Biolabs, Beverly, MA, USA) with *BamHI* and *XhoI* restriction enzyme sites. The recombinant plasmids were transformed into *Escherichia coli* (*E. coli*) DH5α competent cells (Vazyme, Nanjing, China) and incubated on agar plates supplemented with kanamycin for 12 h at 37 °C. Plasmid verification was performed by DNA sequencing (Sangon Biotech Co., Ltd., Shanghai, China). The confirmed recombinant plasmid was named pET28a-*I215L*.

### 2.3. Expression and Purification of Recombinant pI215L (rpI215L) Protein

The 6× His-tagged fusion *I215L* protein was expressed in *E. coli* BL21(DE3) (Vazyme, Nanjing, China). The cells were cultured in Luria-Bertani (LB) medium at 37 °C with shaking at 200 rpm until the optical density at 600 nm (OD600) of the culture was approximately 0.4~0.6. 1 mM isopropyl-β-D-1-thiogalactoside (IPTG) was added to induce the expression of rpI215L protein at 37 °C for 8 h. RpI215L protein expression was examined by sodium dodecyl sulfate-polyacrylamide gel electrophoresis (SDS-PAGE) analysis using cell lysates. To purify rpI215L, bacterial cells were recovered by centrifugation at 4000× *g* for 15 min and resuspended in cold phosphate-buffered saline (PBS). Cells were sonicated (300 W, 6 s bursts with 6 s pauses) for 15–20 min on ice. Soluble proteins were collected in the supernatant after the cell lysates were centrifuged at 12,000× *g* for 30 min, and filtered with a 0.22 µm filter. Nickel column affinity chromatography was performed for His-tagged rpI215L protein purification with HisSep Ni-NTA agarose resin column. The eluted protein was analyzed for purity by SDS-PAGE, and verified by western blot with both anti-His mAbs (Proteintech Group, Inc., Rosemont, IL, USA) and anti-ASFV antibody serum, respectively.

### 2.4. Anti-rpI215L mAbs Preparation

Anti-rpI215L mAbs were prepared by Hybridoma technology as previously described [20]. 6-week-old female BALB/c mice were immunized with 100 µg of purified rpI215L protein emulsified in an equal volume of incomplete Freund’s adjuvant (Sigma-Aldrich (Shanghai) Trading Co., Ltd., Shanghai, China) by subcutaneous injection. The immunizations were performed four times at 2-week intervals. Three days after the last booster immunization, spleen cells were harvested from the immunized mice and fused with SP2/0 myeloma cells. Confluent cells were cultured in Dulbecco’s modified Eagle’s medium (DMEM) containing HAT Supplement (Sigma-Aldrich) and 20% FBS. Supernatants from the confluent cells were collected at 7 days post fusion and detected for anti-rpI215L antibodies by indirect ELISA using wells coated with rpI215L protein. Positive wells were selected and subcloned into single-cell clones three times by limiting dilution. To prepare ascites containing antibodies against rpI215L protein, 500 µL sterilized paraffin was intraperitoneally injected into 10-week-old female BALB/c mice, 7 days after which approximately 1.0 × 10^6^ hybridoma cells were injected into the peritoneal cavity of the mice. Ascites were collected after 7 days and determined by indirect ELISA.

### 2.5. Indirect ELISA

Each well of flat-bottom polystyrene plates was coated with 100 µL of purified rpI215L protein diluted carbonated coating buffer (pH 9.5) to a final concentration of 2 μg/mL at 4 °C overnight. The plates were washed three times with PBST (0.05% Tween in PBS, *v*/*v*) and blocked with 5% skimmed milk in PBS for 2 h at 37 °C. Hybridoma supernatants and ascites were diluted and added into the plates at 100 μg/well and incubated for 1 h at 37 °C. Positive serum obtained from rpI215L protein-immunized mice and negative serum from unimmunized mice (1:1000 dilution) were used as controls. After washing three times with PBST, horseradish peroxidase (HRP)-conjugated goat anti-mouse IgG (Beyotime Biotechnology Co., Ltd., Shanghai, China) (1:5000 dilution) was added as the secondary antibody and incubated for 45 min at 37 °C. Following another wash step, 50 µL of chromogenic substrate solution (TMB) (Beyotime Biotechnology Co., Ltd., Shanghai, China) was added and incubated for 10 min at 37 °C in the dark. 50 µL of 2 M H_2_SO_4_ was used to stop the reaction. OD values were measured at 450 nm using a microplate reader (BioTek Instruments, Winooski, VT, USA).

### 2.6. SDS-PAGE and Western Blot Analysis

Samples were mixed with 5× loading buffer and heated at 100 °C for 10 min. Proteins in each sample were separated by 12.5% SDS-PAGE. For coomassie blue staining analysis, the gels were stained with the staining solution for 10 min, after which the bands were decolorized with decolorization solution and determined. For western blot analysis, protein samples were transferred onto nitrocellulose filter membranes from the SDS-PAGE. After blocking with 5% skimmed milk for 2 h at room temperature, the membranes were washed 5 times with PBST. Primary antibodies, including anti-His mAbs, anti-ASFV antibody serum, or six anti-rpI215L mAbs, were used to incubate the membranes for 2 h at room temperature. Following another 5 times washes, secondary antibodies of HRP-labelled goat anti-mouse or -pig IgG were added and incubated for 1 h at room temperature. After a final wash step, the blots were scanned and analyzed using a digital imaging system.

### 2.7. Indirect Immunofluorescence Assay (IFA)

PAMs seeded in 12-well plates were infected with ASFV (MOI = 0.1) and fixed at 48 h post-infection with 4% paraformaldehyde (PFA) for 30 min at room temperature. Then the fixed cells were permeabilized with 0.2% Triton X-100 in PBS for 10 min at room temperature. 2% BSA in PBS was used to block the permeabilized cells for 3 h at room temperature. Anti-p30 antibody, anti-ASFV antibody positive serum, and the six generated anti-rpI215L mAbs were used, respectively, as primary antibodies to incubate the fixed cells for 2 h at room temperature. Coralite488-conjugated Affinipure Goat Anti-Mouse IgG(H + L) (Proteintech Group, Inc., Rosemont, IL, USA) was used as the secondary antibody to incubate the cells for 50 min at 37 °C. Five washes with PBS were performed after each of the above steps. Finally, the cells were observed and photographed by Zeiss Axio Observer (Carl Zeiss, Jena, Germany). Experiments with live ASFV were performed in the Biosafety Level 3 Laboratory of Lanzhou Veterinary Research Institute.

### 2.8. Monoclonal Antibodies Secretion Stability and Isotype Determination

Hybridoma cells secreting anti-rpI215L mAbs were passaged continuously for 20 generations. Cell supernatants were collected and examined for antibody titers by indirect ELISA every 5 generations. Isotypes of the six anti-rpI215L mAbs were determined with a Mouse Monoclonal Antibody Subtype Identification Kit following the instructions.

### 2.9. Epitope Mapping and Analysis

The antigenic determinant regions of rpI215L were identified with the six generated anti-rpI215L mAbs and the peptide scanning method, where eight overlapping peptides of rpI215L were expressed in *E. coli* BL21(DE3) with the corresponding gene fragments cloned into pET28a plasmid (primers shown in Table 1). Western blot was performed to explore the epitopes recognized by the six mAbs.

The identified B cell epitopes were predicted using DNAStar Protean 7.0 software. The structure of the pI215L protein was predicted by Robetta service (https://robetta.bakerlab.org/ (accessed on 5 April 2023)) and the locations of the epitopes on the pI215L protein were analyzed by PyMOL software 2.5.0.

To verify the conservation of the two epitopes in different ASFV genotypes, sequences of 23 isolates from seven different genotypes were obtained from the Genbank database and aligned using Jalview Version 2.

To explore the reactivity between the six mAbs and the corresponding epitopes in various ASFV genotypes, eight mutants of *I215L* gene, S80P-*I215L*, L67I/F69Y/P76R/S80P-*I215L*, T70I/S80A-*I215L*, L67I/F69Y/P76R-*I215L*, F170C/A174V-*I215L*, F170C/A173V-*I215L*, A174T/S175F-*I215L* and F170C-*I215L* were amplified by fusion PCR with specific primers (Table 2), and the corresponding proteins were expressed and purified as described above.

## 3. Results

### 3.1. Antigen Preparation

**Figure 1:**
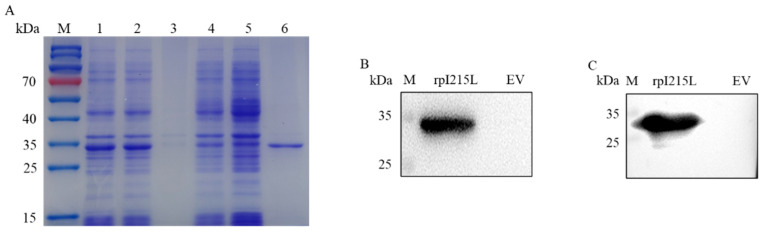
Analysis of rpI215L protein. (**A**) SDS-PAGE analysis of His-tagged rpI215L protein expression, followed by Coomassie brilliant blue stain. M is protein ladder, lane 1, whole bacterial cell lysates after induction with IPTG; lane 2, supernatants of bacterial cell lysates after induction with IPTG; lane 3, precipitates of bacterial cell lysates after induction with IPTG; lane 4, whole bacterial cell lysates with no IPTG induction; lane 5, negative control with pET28a vector; lane 6, the purified rpI215L protein. (**B**) Western blot analysis of rpI215L protein with anti-His tag antibody. (**C**) Western blot analysis of rpI215L protein with anti-ASFV antibody serum.

RpI215L protein was induced and expressed in the *E. coli* BL21(DE3). High-yield rpI215L protein (29.0 kDa) was expressed in a soluble form in the supernatant of the sonicated *E. coli* cell lysates (Figure 1A). After purification with the HisSep Ni-NTA agarose resin column, the purified rpI215L protein was confirmed and showed good reactivities with both anti-His mAb and anti-ASFV antibody serum (Figure 1B,C).

### 3.2. Generation of mAbs against ASFV rpI215L

**Figure 2:**
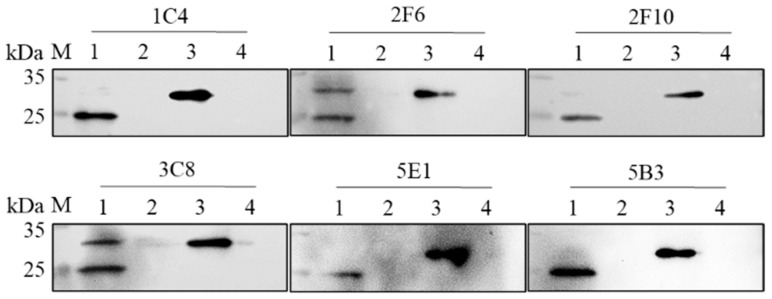
Western blot analysis of anti-rpI215L monoclonal antibodies with PAM-expressed viral pI215L protein and *E. coli*-expressed rpI215L protein. Lane 1, ASFV-infected PAM cell lysates; lane 2, PAM cell lysates; lane 3, purified *E. coli*-expressed rpI215L protein; lane 4, *E. coli* cell lysates (negative control with pET28a vector).

Six hybridoma cell lines, named 1C4, 2F6, 2F10, 3C8, 5E1 and 5B3, against ASFV rpI215L protein were produced. To determine the specificity of the six mAbs, the purified rpI215L protein and the PAMs-expressed viral pI215L protein were analyzed by western blot using 1C4, 2F6, 2F10, 3C8, 5E1, and 5B3 as primary antibodies, respectively, and the six mAbs were shown to specifically recognize both of them (Figure 2). IFA results showed no green fluorescence, indicating that the six mAbs might recognize some specific linearized epitopes tucked inside the structural viral pI215L protein or that the epitopes were occupied by other molecules during virus replication in PAMs (Figure S1).

### 3.3. Monoclonal Antibody Secretion Stability Analysis

1C4, 2F6, 2F10, 3C8, 5E1, and 5B3 hybridoma cell lines were passaged for 20 generations, and cell supernatants of the 5th, 10th, 15th, and 20th generations were collected and examined for antibody titers. Antibody titers in 1C4, 2F10, 3C8, 5E1, and 5B3 hybridoma cell lines were stably maintained at 1:12,800; the mAbs titers in the 2F6 hybridoma cell line slightly increased from P10 to P15 and were maintained at 1:25,600 until P20. 1C4 mAb titer in ascites were 1:12,800, and 2F6, 2F10, 3C8, 5E1 and 5B3 mAbs titers in ascites were 1:25,600 (Table 3).

### 3.4. Monoclonal Antibody Isotype Determination

Monoclonal antibody isotypes were characterized using a Mouse Ig Isotyping Kit (Proteintech Group, Inc., Rosemont, IL, USA). 1C4, 5E1 and 5B3 were found to be IgG1 with λ light chain, 2F6 was IgG2a with κ light chain and 2F10 and 3C8 were IgG1 with κ light chain (Table 4).

### 3.5. RpI215L Epitopes Identification

**Figure 3:**
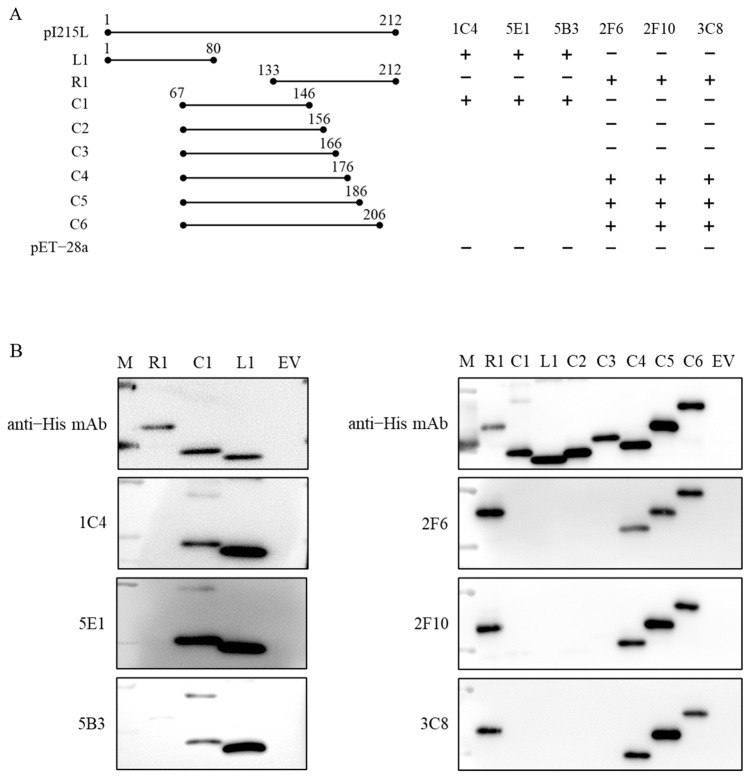
Antigen epitope identification. (**A**) Strategy for the identification of the epitopes within rpI215L protein and reactivity between the truncated rpI215L protein fragments and the six mAbs. +, interaction; −, no interaction. (**B**) Western blot analysis of the reactivity between the truncated rpI215L protein fragments and the six mAbs.

The antigenic epitopes of the rpI215L protein were explored with eight overlapped peptides spanning the rpI215L protein by western blot analysis (Figure 3A). Western blot with L1 (1–80 aa), C1 (67–146 aa) and R1 (133–212 aa) fragments and 1C1, 5E1 and 5B3 mAbs discovered an epitope located at 67LTFTSEMWHPNIYS80 of rpI215L protein. Reactivity analysis between fragments L1, R1, C1 to C6 and 2F6, 2F10, and 3C8 mAbs further revealed an epitope within 167IEYFKNAASN176 (Figure 3B).

### 3.6. Epitope Characteristic Analysis

**Figure 4:**
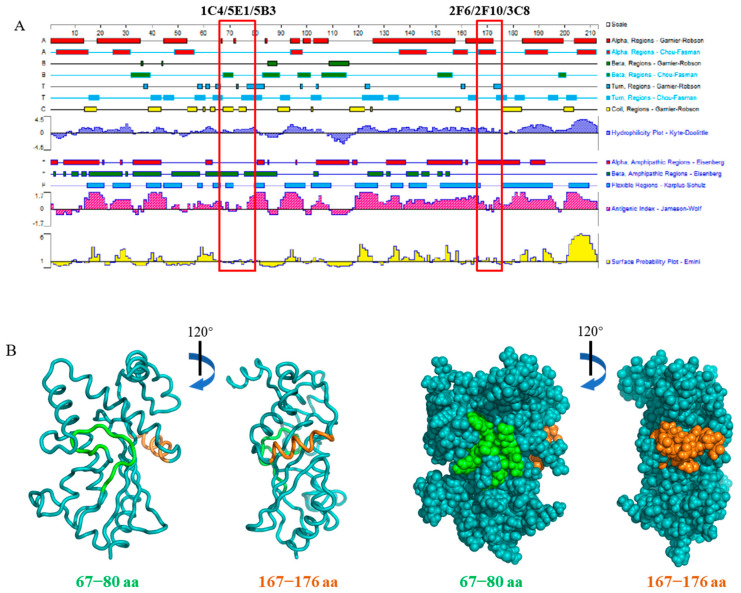
Epitope characteristics analysis. (**A**) Evaluation of the B cell epitopes as antigenic targets by DNAStar Protean 7.0. (**B**) Locations of the B cell epitopes on pI215L protein predicted by PyMOL.

DNAstar Protean 7.0 software prediction of the identified epitopes of pI215L protein showed that 67LTFTSEMWHPNIYS80 was mainly in an irregular structure containing β-sheet, turns, and coils, partially exposed on the surface of the protein, showing good hydrophilicity and antigenicity. 167IEYFKNAASN176 was predicted to be structured as an alpha helix mostly exposed on the surface of the protein with good hydrophilicity and antigenicity (Figure 4A). Further structural analysis by PyMOL supported the DNAstar Protean prediction, 67LTFTSEMWHPNIYS80 was folded at a depression part on the surface of the protein, while 167IEYFKNAASN176 was more exposed at a bulge structure on the protein surface (Figure 4B).

### 3.7. Conservative Analysis of the Epitopes

**Figure 5:**
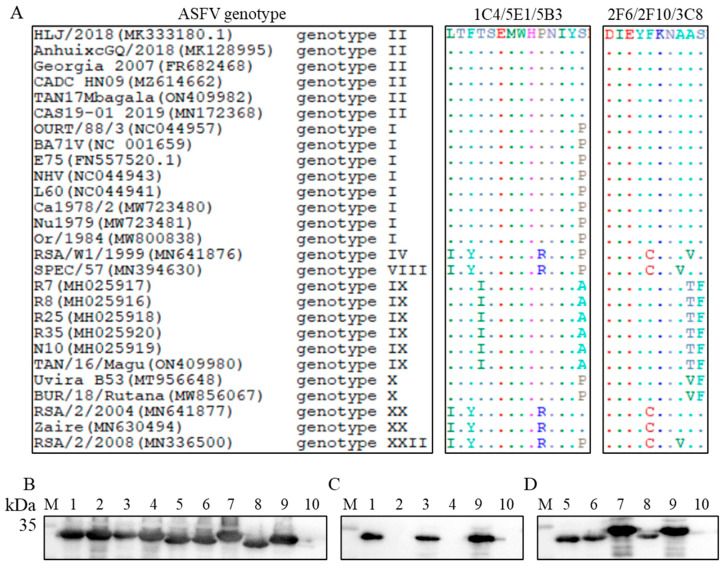
Sequence conservation analysis of the identified epitope among different ASFV genotypes. (**A**) Homology comparison of the identified epitope among 23 ASFV reference strains collected from GenBank. (**B**) Identification of the rpI215L protein mutants with anti-His tag antibody. (**C**) Reactivity of 5B3 mAb against the rpI215L protein and its epitope specific-mutants. (**D**) Reactivity of 2F6 mAb against the rpI215L protein and its epitope specific-mutants. M is protein ladder, lane 1, S80P-rpI215L protein; lane 2, L67I/F69Y/P76R/S80P-rpI215L protein; lane 3, T70I/S80A-rpI215L protein; lane 4, L67I/F69Y/P76R-rpI215L protein; lane 5, F170C/A174V-rpI215L protein; lane 6, F170C/A173V-rpI215L protein; lane 7, A174T/S175F-rpI215L protein; lane 8, F170C-rpI215L protein; lane 9, rpI215L protein; lane 10, negative control with pET28a vector.

As shown in Figure 5, epitope 67LTFTSEMWHPNIYS80 was conserved among ASFV genotype II, and 167IEYFKNAASN176 was conserved among ASFV genotypes I and II (Figure 5A). To further investigate whether the mutations occurring in different genotypes would ruin the reactivity between the six mAbs and the corresponding epitopes, eight mutants of rpI215L protein, S80P-rpI215L, L67I/F69Y/P76R/S80P-rpI215L, T70I/S80A-rpI215L, L67I/F69Y/P76R-rpI215L, F170C/A174V-rpI215L, F170C/A173V-rpI215L, A174T/S175F-rpI215L and F170C-rpI215L were generated and expressed. Western blot analysis showed that the eight mutants of rpI215L protein were correctly expressed and recognized by anti-His tag antibody (Figure 5B). Figure 5C showed that 5B3 mAb recognized rpI215L, S80P-rpI215L, and T70I/S80A-rpI215L but not L67I/F69Y/P76R/S80P-rpI215L and L67I/F69Y/P76R-rpI215L, indicating that it could distinguish genotypes I, II, and IX from genotype IV, VIII, XX, and XXII. Meanwhile, the mutations within 167IEYFKNAASN176 did not block the reactions between 2F6 mAb and the epitope, indicating a general discrimination function of 2F6 against various ASFV genotypes (Figure 5D).

## 4. Discussion

ASF is a highly contagious viral disease leading to terrible economic and production losses to the global pig industry. World Organization for Animal Health (WOAH) takes ASF as a notifiable disease owing to its ability to spread rapidly and cause severe illness. To date, there are few effective commercial vaccines or antiviral drugs available against ASF. Therefore, diagnosis and elimination of the infected animals at an early infection stage is of great significance for ASF prevention and control in affected countries [21]. Serological assays used for antibody detection are widely applied during ASFV surveillance, ASFV infection induces quick and enduring antibody response [22]. MAbs against ASFV major capsid protein p72, the structural and highly immunogenic protein p30, and the type II transmembrane protein p54 which plays a key role in virus morphogenesis and viral infection have mostly been used as targets for the detection of ASFV infection in serological assays [10,23,24,25]. ASFV pI215L protein was abundant in virus-infected PAMs [14] and exhibited good antigenicity as shown in Figure 1C. The potential of pI215L protein as a target for early detection of ASFV infection and its sensitivity compared to p30 and p72 is worth exploring. However, so far, there are still no reports revealing the levels and growth of anti-pI215L antibodies in the serum of ASFV-infected pigs. Further exploration is needed to determine whether pI215L could be a better target for ASFV diagnosis.

In this study, rpI215L protein was first expressed using a prokaryotic-expressing system and identified with anti-ASFV antibody serum. Western blot results showed a strong reactivity between rpI215L protein and anti-ASFV antibody serum, indicating a good antigenicity of pI215L protein, therefore a possible role of pI215L protein as a target for ASFV detection. Six antibodies, 1C4, 5E1 and 5B3 (IgG1/λ type), 2F10 and 3C8 (IgG1/κ type), and 2F6 (IgG2a/κ type) were generated using hybridoma technology. As demonstrated in the stability study, the six hybridoma cell lines stably produced anti-rpI215L protein antibodies at high levels from 1:12,800 to 1:25,600. Western blot analysis showed that all the six mAbs specifically interacted with ASFV pI215L protein. MAbs 2F6 and 3C8 recognized two bands against viral pI215L protein, giving a hint that pI215L protein might be modified and shown as different forms during virus infection in cells. For example, Freitas et al. found that pI215L protein could be mono- and di-ubiquitinated during virus replication [16]. IFA analysis showed no specific fluorescence targeted by the six mAbs, indicating the key amino acids recognized by the mAbs could be tucked inside the protein, functionally modified, or occupied with other molecules during virus infection in cells.

The significance of epitope analysis is increasing as epitopes could not only be used for rational vaccine design but also serve as targets for antiviral development. As a multifunctional protein that is essential for ASFV replication and displays good antigenicity, the functional active sites or epitopes of pI215L protein have not been reported yet. In the present study, two novel epitopes, 67LTFTSEMWHPNIYS80 and 167IEYFKNAASN176 were identified with the six mAbs. Further analysis indicated a good hydrophilicity and antigenicity of the epitopes, as well as a partial exposure of the epitopes on the surface of the pI215L protein. This might explain the negative reactivity of the generated six mAbs against viral pI215L protein in PAMs by IFA as the key amino acids recognized by the mAbs might be folded inside the fully structured protein. On the other hand, as western blot results showed a different position of the viral pI215L protein on SDS-PAGE gel from the *E. coli*-expressed rpI215L protein, it is suggested that the epitopes of the viral protein might be specifically modified with other molecules in ASFV-infected PAMs, which impaired the reaction between mAbs and the epitopes. Sequence conservation analysis of the I215L gene with 23 ASFV strains belonging to 7 genotypes revealed a series of mutations (L67I, F69Y, T70I, P76R, S80P, S80A, F170C, A173V, A174V, A174T and S175F) within the two identified epitopes among different ASFV genotypes. Eight rpI215L protein mutants were next generated for western blot analysis, and the results showed that the mutations within 167IEYFKNAASN176 epitope did not block the interaction between 2F6 mAb and rpI215L proteins, while L67I/F69Y/P76R/S80P within 67LTFTSEMWHPNIYS80 broke down the interaction between 5B3 mAb and rpI215L protein. These results suggested that 2F6 mAb could be used in a wide application for various ASFV genotypes surveillance and 5B3 mAb could be used to distinguish genotypes I, II, and IX from genotypes IV, VIII, XX, and XXII, which needs to be further studied in the future.

In summary, six monoclonal antibodies 1C1, 2F6, 2F10, 3C8, 5E1, and 5B3 against ASFV pI215L protein were generated in this study, which could be used in both ASFV surveillance and different genotype distinguishing, and two novel epitopes were identified. These findings not only provide tools of monoclonal antibodies for functional study of *I215L* but also contribute to serological diagnosis and vaccine development.

## Figures and Tables

**Table 1:** Primers used for I215L truncations construction.

Fragments	Primers	Sequences (5′-3′)	Position (Amino Acid)
pI215L	PF	CGCGGATCCATGGTTTCCAGGTTTTTAAT	1–212 aa
	PR	CCGCTCGAGCTCATCATCCTCCTCTTCTT	
L1	L1-PF	CGCGGATCCATGGTTTCCAGGTTTTTAAT	1–80 aa
	L1-PR	CCGCTCGAGAGAGTAAATATTAGGATGCC	
R1	R1-PF	CGCGGATCCAAAAGCTACCGTAAACTC	133–212 aa
	R1-PR	CCGCTCGAGCTCATCATCCTCCTCTTCTT	
C1	C1-PF	CGCGGATCCTTAACATTCACCTCTGAAAT	67–146 aa
	C1-PR	CCGCTCGAGTGATTCCAAATCCTCTTTAT	
C2	C2-PF	CGCGGATCCTTAACATTCACCTCTGAAAT	67–156 aa
	C2-PR	CCGCTCGAGTTTGACAGTCCTTTTAACT	
C3	C3-PF	CGCGGATCCTTAACATTCACCTCTGAAAT	67–166 aa
	C3-PR	CCGCTCGAGGTCTTCTGGTGAACACT	
C4	C4-PF	CGCGGATCCTTAACATTCACCTCTGAAAT	67–176 aa
	C4-PR	CCGCTCGAGATTGGATGCAGCATTTT	
C5	C5-PF	CGCGGATCCTTAACATTCACCTCTGAAAT	67–186 aa
	C5-PR	CCGCTCGAGTTCATAAGCATCACTGG	
C6	C6-PF	CGCGGATCCTTAACATTCACCTCTGAAAT	67–206 aa
	C6-PR	CCGCTCGAGTTCATCCTCATCATCAT	

**Table 2:** Primers used for I215L mutant construction.

Fragments	Primers	Sequences (5′-3′)
S80P-*I215L*	PF	CTAATATTTACCCTGATGGAAAAC
	PR	CATAGTTTTCCATCAGGGTAAATA
L67I/F69Y/P76R/ S80P-*I215L*	PF	CCCAGAATAACATACACCTCTGAAATGTGGCATCGTAATATTTACCCTGATGGA
	PR	CCATCAGGGTAAATATTACGATGCCACATTTCAGAGGTGTATGTTATTCTGGGTG
T70I/S80A-*I215L*	PF	GATTAACATTCATCTCTGAAATGTGGCATCCTAATATTTACGCTGATGGAAA
	PR	GTTTTCCATCAGCGTAAATATTAGGATGCCACATTTCAGAGATGAATGTTA
L67I/F69Y/P76R-*I215L*	PF	CACCCAGAATAACATACACCTCTGAAATGTGGCATCGTAATATTTAC
	PR	GAGTAAATATTACGATGCCACATTTCAGAGGTGTATGTTATTCTGG
F170C/A174V-*I215L*	PF	CATAGAATATTGTAAAAATGCTGTATCCAATG
	PR	GGTGGAACATTGGATACAGCATTTTTACAATATTC
F170C/A173V-*I215L*	PF	CATAGAATATTGTAAAAATGTTGCATCCAA
	PR	GAACATTGGATGCAACATTTTTACAATATTCT
A174T/S175F-*I215L*	PF	GAATATTTTAAAAATGCTACATTCAATGTTCCAC
	PR	GTATTGGTGGAACATTGAATGTAGCATTTTTAA
F170C-*I215L*	PF	CATAGAATATTGTAAAAATG
	PR	GCAGCATTTTTACAATATTC

**Table 3:** Monoclonal antibody titers in hybridoma cell supernatant and ascites.

Monoclonal Antibody	Hybridoma Cell Supernatant Antibody Titers				Ascites Titers
	P5	P10	P15	P20	
1C4	1:12,800	1:12,800	1:12,800	1:12,800	1:12,800
2F6	1:12,800	1:12,800	1:25,600	1:25,600	1:25,600
2F10	1:12,800	1:12,800	1:12,800	1:12,800	1:25,600
3C8	1:12,800	1:12,800	1:12,800	1:12,800	1:25,600
5E1	1:12,800	1:12,800	1:12,800	1:12,800	1:25,600
5B3	1:12,800	1:12,800	1:12,800	1:12,800	1:25,600

**Table 4:** Monoclonal antibody isotype identification.

Monoclonal Antibody	Hybridoma Cell Supernatant Antibody Titers							
	IgG1	IgG2a	IgG2b	IgG2c	IgG3	IgM	Igκ	Igλ
1C4	**3.186**	0.208	0.205	0.105	0.126	0.135	0.130	**2.026**
2F6	0.225	**2.693**	0.230	0.245	0.196	0.301	**1.951**	0.246
2F10	**2.042**	0.196	0.154	0.201	0.186	0.215	**1.704**	0.231
3C8	**3.012**	0.241	0.235	0.199	0.261	0.186	**2.013**	0.195
5E1	**2.986**	0.199	0.216	0.236	0.208	0.215	0.227	**1.987**
5B3	**2.045**	0.241	0.237	0.236	0.202	0.189	0.256	**2.154**

## Data Availability

All data created in this study has already been shown in the manuscript. No more new data is available.

## Supplementary Materials

The following supporting information can be downloaded at: https://www.mdpi.com/article/10.3390/v15102081/s1, Figure S1: The reactivity of the monoclonal antibodies detected by IFA on ASFV–infected PAMs.
