# Supplementary material for: Novel Epitope Mapping of African Swine Fever Virus pI215L Protein Using Monoclonal Antibodies
Source: Viruses. 2023 Oct 12;15(10):2081. doi: 10.3390/v15102081 (PMC10612046; doi:10.3390/v15102081)
Supplement: Supplementary file 1 [file viruses-15-02081-s001.zip › viruses-2661024-supplementary.pptx]

## Slide 1
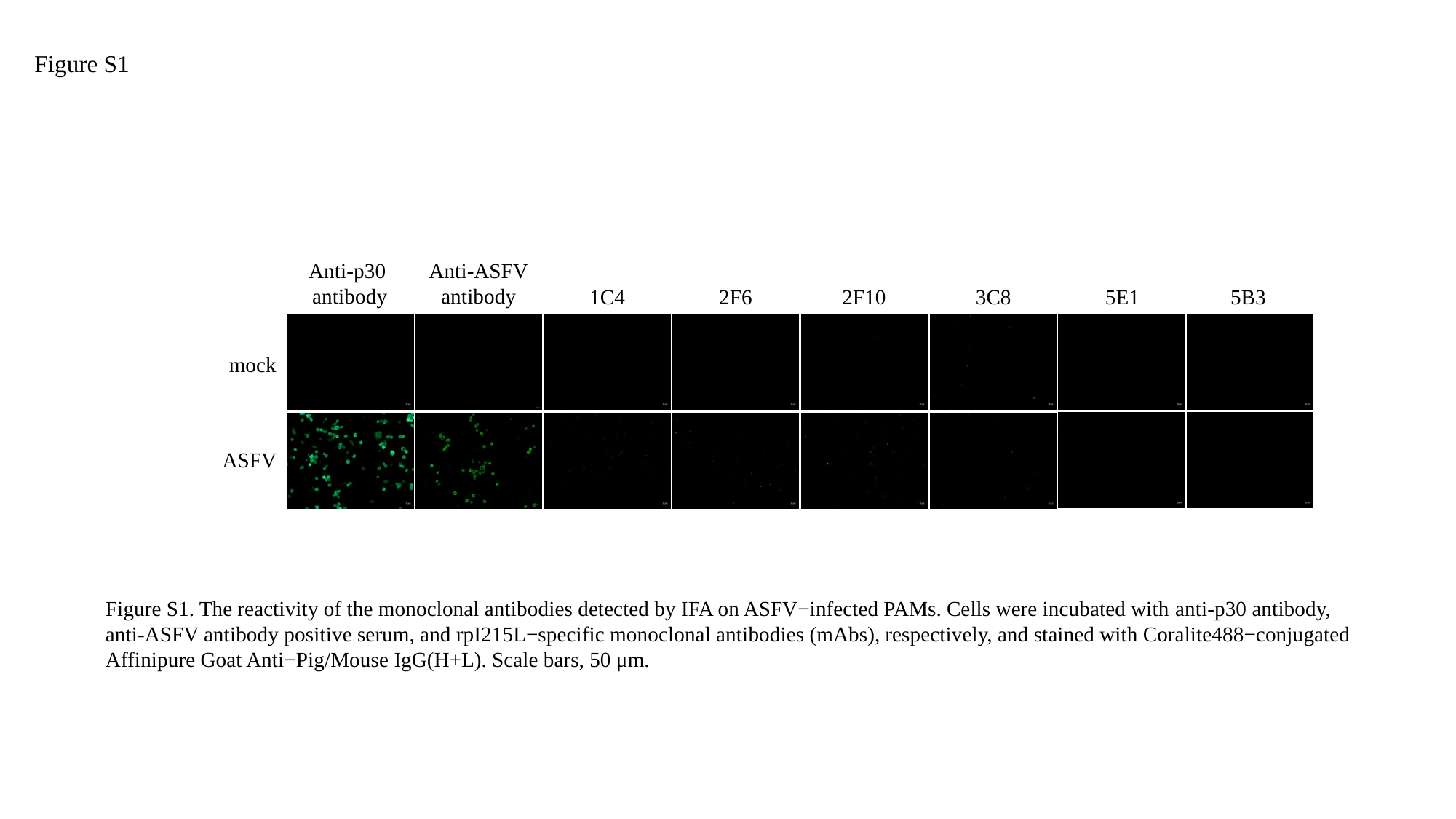

Figure S1
Anti-p30
antibody
Anti-ASFV
antibody
1C4
2F6
2F10
3C8
5E1
5B3
mock
ASFV
Figure S1. The reactivity of the monoclonal antibodies detected by IFA on ASFV−infected PAMs. Cells were incubated with anti-p30 antibody, anti-ASFV antibody positive serum, and rpI215L−specific monoclonal antibodies (mAbs), respectively, and stained with Coralite488−conjugated Affinipure Goat Anti−Pig/Mouse IgG(H+L). Scale bars, 50 μm.
